# Pharmaceutical care-based interventions in type 2 *diabetes mellitus* : a systematic review and meta-analysis of randomized clinical trials

**DOI:** 10.31744/einstein_journal/2020RW4686

**Published:** 2020-01-27

**Authors:** Marcel Nogueira, Leonardo Jun Otuyama, Priscilla Alves Rocha, Vanusa Barbosa Pinto

**Affiliations:** 1 Hospital das Clínicas Faculdade de Medicina Universidade de São Paulo São PauloSP Brazil Hospital das Clínicas, Faculdade de Medicina, Universidade de São Paulo, São Paulo, SP, Brazil.

**Keywords:** Diabetes mellitus, type 2, Pharmaceutical services, Pharmacy service, hospital, Pharmacists

## Abstract

**Objective:**

To investigate the impact of pharmaceutical care-based interventions on type 2 *diabetes mellitus* .

**Methods:**

PubMed^®^, Cochrane and Web of Science data bases were searched for randomized controlled clinical trials. Studies evaluating pharmaceutical care-based interventions in type 2 *diabetes mellitus* published between 2012 and 2017 were included. Glycated hemoglobin was defined as the primary endpoint; blood pressure, triglycerides and cholesterol as secondary endpoints. The random effects model was used in meta-analysis.

**Results:**

Fifteen trials involving 2,325 participants were included. Meta-analysis revealed considerable heterogeneity (I^2^>97%; p<0.001), reduction in glycated hemoglobin (-1.07%; 95%CI: -1.32; -0.83; p<0.001), glucose (-29.91mg/dL; 95%CI: -43.2; -16.6; p<0.001), triglyceride (19.8mg/dL; 95%CI: -36.6; -3.04; p=0.021), systolic blood pressure (-4.65mmHg; 95%CI: -8.9; -0.4; p=0.032) levels, and increased HDL levels (4.43mg/dL; 95%CI: 0.16; 8.70; p=0.042).

**Conclusion:**

Pharmaceutical care-based clincal and education interventions have significant impact on type 2 *diabetes mellitus* . The tools Summary of Diabetes Self-Care Activities and the Morisky Medication Adherence Scale may be useful to monitor patients.

## INTRODUCTION

Type 2 *diabetes mellitus* (T2DM) is characterized by pre- and postprandial hyperglycemia, combined with relative insulin insufficiency resulting from inadequate insulin secretion and low insulin sensitivity.^1^ Type 2 DM is a chronic disease with alarming growth rates in several countries, and is expected to become a serious health concern over the next decades.^2 , 3^ Type 2 is the most common form of diabetes and has been on the rise alongside cultural and social changes. Diabetes currently affects approximately 415 million people worldwide, with a global prevalence of 8.3% among adults; this number is expected to rise to 592 million, in 2035. Type 2 DM is thought to affect 14.3 million adult individuals in Brazil. Aside from individual costs associated with medical treatment of diabetes, the disease places a major economic burden on countries and respective health systems due to diabetes-related complications.^4^

Ongoing education is useful for patients,^1^ since diabetes management is complex and involves glucose monitoring, adherence to treatment, physical activity and dietary changes.^5^ Glycated hemoglobin (HbA1c) reflects blood glucose levels over the last 120 days and is the gold standard biomarker for diabetes control assessment and prediction of severe complications.^6^ According to current guidelines, blood glucose levels should be close to normal in order to prevent or delay complications.^1^ However, lack of adherence to treatment is common and may impact glycemic control, with increased mortality rates.^7^ Inadequate glycemic control is associated with increased risk of cardiovascular disease, neuropathy, retinopathy, nephropathy and hospitalization.^6 , 8^ Adherence to treatment is vital to fully benefit from therapeutic regimens. Approximately 20% to 50% of patients with chronic diseases report less than optimal adherence to drug therapy, which hinders effectiveness of treatment.^9 , 10^ Also, high glycemic indices have been reported in patients with poor adherence to treatment.^11 , 12^

Several factors have been associated with non-adherence to treatment, including social and economic aspects, pharmacotherapy complexity and patients’ beliefs regarding drugs.^13^ As pharmacotherapy specialists, clinical pharmacists contribute to patient care by providing individual guidance (alone or with other health professionals), assisting in planning and monitoring of therapeutic strategies aimed to improve the pathological conditions, treatment and adherence by a process entitled “pharmaceutical care”.^14 , 15^ This may be defined as “responsible provision of pharmacotherapy to achieve outcomes associated with improved quality of life for patients.”^16^

Identification of drug-related problems is another a vital aspect of pharmaceutical care aimed to prevent events that may impact on treatment outcomes, such as pharmacotherapy effectiveness, adverse reactions and polypharmacy, which are common among diabetic patients.^17 - 21^ Pharmaceutical care also provides drug-related information to help patients understand pharmacotherapy risks and benefits, so as to improve adherence to treatment and clinical outcomes.^13^

However, decision making in diabetic patient care has not yet been fully understood.^22^ There is a need to determine which pharmacist interventions are currently applicable in clinical practice. Also, only one meta-analysis of studies investigating pharmaceutical care in T2DM has been published to date.^23^ This study examined the hypothesis that pharmaceutical care would make significant contributions to T2DM control, particularly regarding HbA1c reduction in affected patients.

## OBJECTIVE

To investigate the impact of pharmacist interventions on glycemic control in type 2 *diabetes mellitus* patients and other endpoints, such as blood pressure, triglycerides and cholesterol levels.

## METHODS

### Search strategy

A systematic review of randomized controlled clinical trials investigating the impact of pharmacist interventions in T2DM management was used as a reference for study selection and identification of new studies.^24^ The following databases were searched: PubMed^®^, Cochrane Central Register of Controlled Trials and Web of Science. Search strategies are detailed in appendix A.

### Date of publication

Studies published between January 2012 and December 2014 were selected. Results of new searches were limited to articles published between January 2015 and October 2017.

### Study selection

Preferred Reporting Items for Systematic Reviews and Meta-Analyses (PRISMA) recommendations were used for systematic review and meta-analysis.^25^ Studies with the following characteristics were selected: population comprising participants aged 18 years or older, with a diagnosis of T2DM; health team comprising one pharmacist capable of providing pharmaceutical care via clinical and/or educational interventions for management of participants with T2DM; comparing standard medical, nursing, and community pharmacy care, and defining HbA1c as the primary endpoint, and systolic (SBP) and diastolic blood pressure (DBP), triglycerides (TG) and cholesterol (low – LDL- and high density – HDL - lipoprotein) levels and adherence to treatment as secondary endpoints; conducted in outpatient clinics, hospitals and community pharmacies; prospective randomized controlled clinical trial design; published in English.

Studies conducted exclusively with participants suffering from type 1 *diabetes mellitus* , *diabetes insipidus* or gestational diabetes, involving interventions based exclusively on educational programs or leaflets, defining fasting blood glucose levels as primary endpoint, retrospective studies and non-randomized controlled clinical trials were excluded.

### Risk of bias assessment

The Cochrane collaboration tool was used to assess selection, performance, detection, attrition, reporting and other biases. Selected studies were classified as having low, high or unclear risk of bias.

### Data extraction

Selected studies were individually evaluated by a single author. Data were extracted in standard format using Microsoft Excel, and consisted of study characteristics, participant characteristics, pharmacist interventions carried out and outcome measures.

### Statistical analysis

Statistical analyses were conducted using software (STATA 13; Statacorp, Texas, USA). The random effects model was selected to account for heterogeneity of studies included in the meta-analysis; I^2^ was calculated to assess the magnitude of heterogeneity between studies (I^2^ >50% and I^2^ >75% indicate substantial and considerable heterogeneity, respectively). The χ^2^ test was used to assess significance of heterogeneity (p<0.10).

### Outcome measures

Outcome measures were defined as changes between the Intervention and the Control Group. Primary (HbA1c) and secondary (SBP, DBP, fasting glucose, TG, LDL and HDL cholesterol levels) endpoint measures were expressed as mean differences and 95% confidence intervals (95%CI). The findings were expressed using conventional measures: percentage for HbA1c; mmHg for SBP and DBP; and mg/dL for TG, LDL and HDL. The level of significance was set at 5%. Whenever results extracted from selected studies were expressed as mmol/L, these were converted to mg/dL.

## RESULTS

### Study selection

The systematic used as reference for this study yielded 36 clinical trials.^24^ Of these, 25 were excluded due to non-conformity with selected time, and 11 were rated eligible. Four out of 11 eligible studies failed to define HbA1c as the primary endpoint. The final sample comprised seven studies.^21 , 26 - 31^

**Figure 1:**
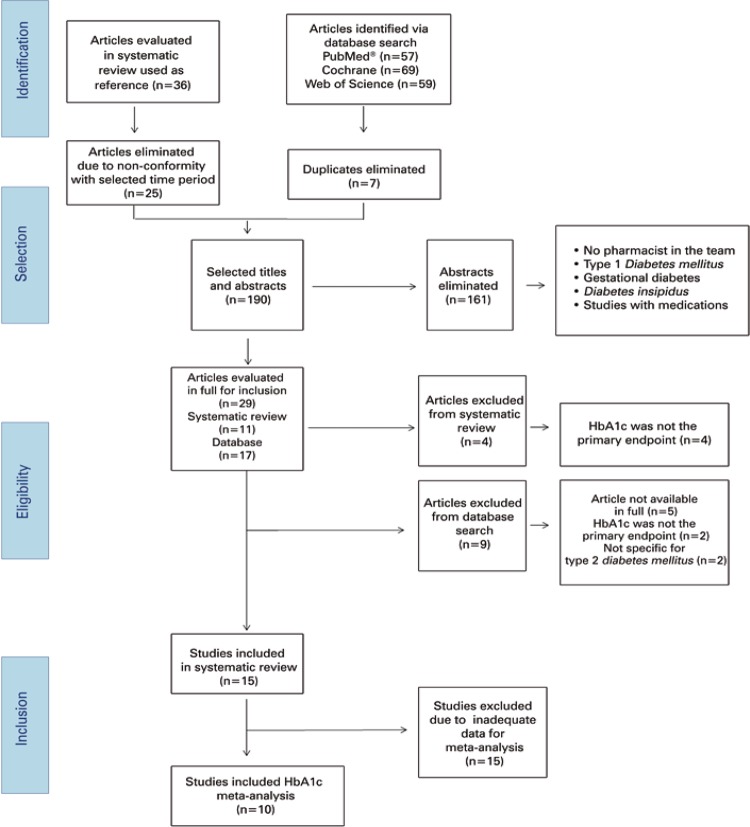
Study selection flowchart HbA1c: Glycated hemoglobin.

The database search strategy adopted yielded 185 results. Seven duplicates were eliminated in the screening process. Following analysis of title and abstract of 161 articles, 17 were selected. Of these, five were excluded since they were not available in full text; two for not defining HbA1c as the primary endpoint and two for not being specific for T2DM. The remaining eight articles were included in the analysis. Overall, 15 articles were included in the systematic review and 10 in the meta-analysis ( Figure 1 ).

### Study characteristics

Studies included in the systematic review were conducted in outpatient clinics and community pharmacies of different countries, as follows: three in Brazil, three in China, one in Malaysia, one in the United Kingdom, one in Singapore, one in Taiwan, one in Iran, one in Iraq, two in Jordan, and one in the Turkish Republic of Northern Cyprus. Experimental design was randomized controlled in all cases and one study was multicenter. Follow-up ranged from 3 to 12 months (mean 7.9 months). Clinical trials included participants with HbA1c ranging from 6.5% to 9%. Detailed description of studies included is given in table 1 .

**Table 1:** Characteristics of studies included

Reference	Settings	Population	Characterization of the sample	Follow-up	Interval of intervention	Pharmacist interventions	Control	Clinical endpoint measures
Mahwi et al.^(21)^	Diabetes center in Iraq	Type 2 diabetics aged 30 to 80 years	n=65/65* Lost to follow-up: 3/4* Age: 52.0±7, 86/53.4±10.81*^†^ Sex: 71.0%/67.2%* female	3 months	Monthly	Adherence, drug-related problems	Standard care by medical team	HbA1c, fasting glucose and drug-related problems, adherence
Jarab et al.^(26)^	Diabetes outpatient service of a teaching hospital in Jordan	Type 2 diabetics aged ≥18 years, HbA1c ≥7.5%	n=85/86* Lost to follow-up: 8/7* Age: 63.4±10.1/65.3±9.2*^†^ Sex: 42.4%/44.2%* female	6 months	8 weeks (follow-up telephone calls)	Patient education, drug-related problems, follow-up telephone calls, self-care	Standard care by medical and nursing teams	HbA1c, adherence, blood pressure, TC, HDL, LDL, TG, BMI, self-care (SDSCA)
Wishah et al.^(27)^	Diabetes outpatient service of a teaching hospital in Jordan	Type 2 diabetics aged ≥18 years, HbA1c ≥6.5%, use of oral hypoglycemic agent	n=52/54* Lost to follow-up: 2/3* Age: 52.9±9.6/53.2±11.2*^†^ Sex: 61.5%/51.9%* female	6 months	Not informed	Patient education, drug- related problems, self-care	Standard care by medical and nursing teams	HbA1c, blood pressure, TC, HDL, LDL, TG, BMI, fasting glucose, self-care (SDSCA)
Chung et al.^(28)^	Teaching hospital in Malaysia	Type 2 diabetics aged ≥21 and <75 years, HbA1c ≥8%, use of oral hypoglycemic agent	n=120/121* Lost to follow-up: NR Age: 59.7±9.5/58.5±8.3*^†^ Sex: 58.3%/53.7%* female	12 months	3 to 4 months with monthly follow-up telephone calls	Medication review, diabetes education	Usual pharmacy services	HbA1c, fasting glucose, adherence
Ali et al.^(29)^	Community pharmacies in the United Kingdom	Type 2 diabetics aged ≥18 years, HbA1c ≥7%, use of oral hypoglycemic agent	n=25/23* Lost to follow-up: 2/0* Age: 66.4±12.7/66.8±10.2*^†^ Sex: 43.5%/56.5%* male	12 months	Monthly in the first 2 months, then every 3 months	Medication review, patient education, referral to other healthcare professionals	Standard care by medical and nursing teams and community pharmacies	HbA1c, fasting glucose, blood pressure, LDL, HDL, TG, BMI, DQoL, HRQoL, adherence, diabetes knowledge test (DKT), SIMS
Mourão et al.^(30)^	Primary Care Units in Brazil	Type 2 diabetics aged ≥18 years, HbA1c ≥7%, post-prandial glucose ≥180mg/dL, use of oral hypoglycemic agent	n=65/64* Lost to follow-up: 12/9* Age: 60.0±10.2/61.3±9.9*^†^ Sex: 68.0%/66.0%* female	12 months	Monthly	Drug-related problems, diabetes education	Standard care	HbA1c, blood pressure, LDL, HDL, TG, BMI, drug-related problems
Chan et al.^(31)^	Diabetes clinic of a public hospital in Hong Kong	Type 2 diabetics aged ≥18 years, HbA1c ≥8%, polypharmacy, use of oral hypoglycemic agent	n=51/54* Lost to follow-up: 0/0* Age: 63.2±9,5/61.74±11.2*^†^ Gender: 58.8%/51.9%* male	9 months	Not reported	Patient education, drug related problems	Standard care by medical team	HbA1c, blood pressure, LDL, HDL, TG, BMI, adherence, cardiovascular risk, cost-effectiveness
Korcegez et al.^(32)^	Diabetes outpatient service in a public hospital in Turkish Republic of Northern Cyprus	Type 2 diabetics diagnosed at least 6 months prior to the study, HbA1c >7% and use of oral hypoglycemic agent	n=75/77* Lost to follow-up: 4/3* Age: 61.80±10.38/62.22±9.54*^†^ Sex: 77.3%/74.0%* female	12 months	2 months	Patient education, drug-related problems, self-care	Standard care by medical and nursing teams	HbA1c, blood pressure, TC, HDL, LDL, TG, BMI, fasting glucose, abdominal circumference, self-care (SDSCA)
Siaw et al.^(33)^	Four outpatient services in Singapore	Type 2 diabetics aged ≥21 years, HbA1c ≥7%, polypharmacy and multiple comorbidities	n=214/197* Lost to follow-up: 3/1* Age: 59.2±8.2/60.1±8.1*^†^ Sex: 52.3%/60.9%* male	6 months	4 to 6 weeks	Patient education, drug-related problems, insulin dose adjustment based on symptoms (SIGN algorithm), follow-up telephone calls	Standard care by medical team	HbA1c, systolic blood pressure, LDL, TG, quality of life (PAID), satisfaction with treatment (DTSQ), use of health services, economic analysis
Cani et al.^(34)^	Diabetes outpatient service at a teaching hospital in São Paulo	Type 2 diabetics aged ≥45 years, HbA1c ≥ 8.0%, use of insulin	n=37/41* Lost to follow-up: 3/5* Age: 61.91±9.58/61.58±8.14*^†^ Sex: 34.0%/36%* male	6 months	Not informed	Patient education, drug-related problems, insulin administration technique	Standard care	HbA1c, knowledge about diabetes, knowledge about drugs, adherence, insulin administration technique, glucose monitoring, quality of life (QoL)
Aguiar et al.^(35)^	University secondary care hospital in São Paulo	Type 2 diabetics diagnosed at least 6 months prior to the study, HbA1c ≥7%, aged between 40 and 79 years, use of oral antidiabetic agent	n=36/37* Lost to follow-up: 0/0* Age: 61.1.6±7.9/62.4±8.2*^†^ Sex: 69.4%/64,9%* female	12 months	2 to 6 months (depending on glucose levels) and follow-up telephone calls between visits	Patient education, drug- related problems, insulin administration technique, follow-up telephone calls	Standard care by medical and nursing teams	HbA1c, blood pressure, LDL, adherence
Chen et al.^(36)^	Nantou Hospital, in Taiwan	Type 2 diabetics aged ≥65 years, with HbA1c ≥9%	n=50/50* Lost to follow-up: NR Age: 72.16±6.6/72.76±5.9*^†^ Sex: 50%/50%* male and female	6 months	Monthly follow-up telephone calls	Patient education, drug-related problems, insulin administration technique, referral to other professionals, follow-up telephone calls	Standard care	HbA1c, fasting glucose, percentage of hospitalizations, economic analysis
Jahangard-Rafsanjani et al.^(37)^	Community pharmacy in Teheran, Iran	Type 2 diabetics with HbA1c >7% and use of oral hypoglycemic agent	n=51/50* Lost to follow-up: 6/10* Age: 57.6±8.3/55.9±8.7*^†^ Sex: 25%/26% female	5 months	Monthly	Patient education, drug- related problems, follow-up telephone calls, self-care	Standard care by medical team	HbA1c, BMI, blood pressure, adherence, self-care (SDSCA)
Xin et al.^(38)^	Tongde Hospital, Hangzhou province, China	Type 2 diabetics aged ≥18 years, no use of insulin over the last 18 months	n=120/120* Lost to follow-up: 6/7* Age: 58.8±14.4/59.2±14.2*^†^ Sex: 51.8%/50.4%* male	12 months	Not informed	Patient education, insulin administration technique, follow-up telephone calls	Standard care by medical team	HbA1c, adherence
Shao et al.^(39)^	University hospital in Nanjing province, China	Type 2 diabetics aged ≥18 years, HbA1c ≥7%, diagnosed 3 months before or earlier, and use of oral hypoglycemic agent	n=20/120* Lost to follow-up: 20/21* Age: 58.86±10.59/59.20±10.34*^†^ Sex: 51%/475%* male	6 months	2 months (in-person) and monthly follow-up telephone calls	Patient education, follow-up telephone calls	Standard care by medical team	HbA1c, adherence, blood pressure, TC, HDL, LDL, TG, BMI, fasting glucose

* relation between intervention and Control Group; ^†^ age in years (mean±standard deviation).

HbA1c: glycated hemoglobin; TC: total cholesterol; HDL: high density lipoprotein; LDL: low density lipoprotein; TG: triglycerides; BMI: body mass index; SDSCA: Summary of Diabetes Self-Care Activities Questionnaire; NR: not reported; DQoL: Diabetes Quality of Life Brief Clinical Inventory; HRQoL: Health-Related Quality of Life; DKT: Diabetes Knowledge Test; SIMS: Satisfaction with Information Received About Medicines; PAID: Problem Areas in Diabetes Questionnaire; DTSQ: Diabetes Treatment Satisfaction Questionnaire; QoL: Quality of Life.

### Risk of bias

As regards selection bias, random sequence generation was thought to be adequate in most studies (10/15; 66.7%). Randomization strategy was not reported in four studies (4/15; 26.7%). High risk of bias was observed in one study (1/15; 6.7%) in which participants were randomized according to medical record number.^32^ Fourteen studies (14/15; 93.4%) failed to describe methods used to conceal random allocation sequences, and only one study reported auditing to ensure allocation concealment (multicenter study).^33^

As regards performance bias, no studies reported blinding to pharmacist’s activities, and exchange of information between participants may have occurred in 14 studies conducted in a single setting, except the multicenter study by Siaw et al *.* ^33^ That trial was thought to involve high risk of bias regarding blinding of participants and professionals, since participants in the Control Group were able to consult pharmacists, if required.^34^

As regards detection bias, only one study reported blinding of raters, who were therefore unaware of groups being evaluated.^35^ The remaining studies were thought to have unclear risk of bias, given measures employed to assess clinical outcomes were not described.

Attrition and reporting biases were limited to one study that retained high risk of bias due to lack of description of one of the secondary endpoints.^27^

**Figure 2:**
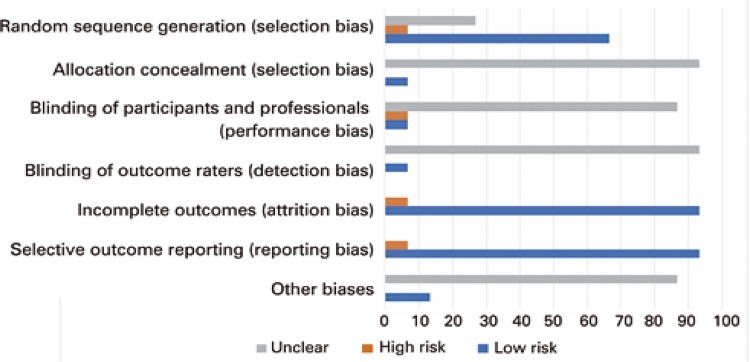
Risk of bias

As for other biases, only two studies were thought to be free from other sources of bias (2/15; 13.4%). Thirteen studies included in this systematic review and meta-analysis (13/15; 86.7%) were thought to have unclear risk of bias, given limitations presented by authors were deemed insufficient to estimate whether significant risk of bias might impact participant outcomes ( Figure 2 and Appendix B).

### Population characteristics

Studies included in this systematic review involved 2,325 participants, with samples ranging from 36 to 214 participants. The proportion of male participants ranged from 22% to 75%. Mean age ranged from 52 to 72 years. Mean baseline HbA1c levels corresponded to 9.06% and 8,79% in the Intervention and Control Group, respectively. Aside from T2DM, most studies included emphasized multiple comorbidities related to secondary clinical endpoints, such as dyslipidemia, hypertension and obesity ( Table 1 ).

### Pharmacist interventions

Interventions were conducted during in-person pharmaceutical visits or via follow-up telephone calls (7/15; 46.7%). However, details of interventions conducted via follow-up telephone calls were not provided in all studies. All clinical trials emphasized patient education activities (15/15; 100%) and collaboration with medical teams (14/15; 93.4%). However, when search terms “drug-related problems” (12/15; 80.0%) or “medication review” (2/15; 26.7%) were used, instruments applied failed to be described. Two studies^21 , 31^ defined frequency of drug-related problems as a clinical outcome of participants, and only two^30 , 35^ described the use of Pharmacotherapy WorkUp^40^ for the same purpose.

“Drug related problems” and “medication review *”* tools were used to identify and solve drug-related problems during patient follow-up. These tools comprise key components of pharmacist interventions, such as: Feedback – recommendations regarding pharmacological treatment forwarded to medical teams in order to solve problems identified and optimize pharmacotherapy (adding, replacing or discontinuing medications, as well as dose adjustments). Follow-up telephone calls – pharmaceutical advice provided over the phone and assessment of adverse events. Patient education – educational interventions related to diabetes and its treatment for improved patient adherence, such as providing information about medications, adverse reactions, administration route and storage (particularly insulin), training aimed at recognition and correction of hypoglycemia, lifestyle changes (smoking cessation, alcoholism, proper diet and foot inspection), self-care promotion (glucose monitoring). All patients received verbal instructions; educational materials, such as leaflets, were also provided in some cases.

In one study, the clinical pharmacist was allowed to adjust insulin doses in naïve patients based on hypoglycemia signs/symptoms. An algorithm was validated for that purpose.^33^

In two studies^29 , 36^ (2/15, 26.7%) participants were referred to other professionals, such as dieticians and nurses.

The Control Group consisted of standard care provided in outpatient settings and community pharmacies, excluding interventions provided by clinical pharmacists, or diabetes education provided by other healthcare professionals, such as physicians and nurses ( Table 1 ).

### Adherence

Adherence to treatment was a clinical endpoint in ten studies. The Morisky Medication Adherence Scale (MMAS-8 and MMAS-4) was used to assess participant adherence at baseline and at the end of the follow-up period. Significant improvement in adherence in the Intervention Group as compared to the Control Group was reported in all studies (p<0.05).

## Meta-analysis

### Heterogeneity analysis

Ten studies were included in the meta-analysis,^21 , 27 - 29 , 32 , 34 , 36 - 39^ all of them with high heterogeneity for all endpoints (I^2^ 97% to 99%; p<0.001). The efficacy of pharmaceutical care to promote reduction of SBP, HbA1c, fasting glucose and TG levels and increase of HDL levels was demonstrated in all studies, in spite of significant heterogeneity. However, the outcomes LDL levels and DBP had no statistically significant differences.

## Glycated hemoglobin levels

**Figure 3:**
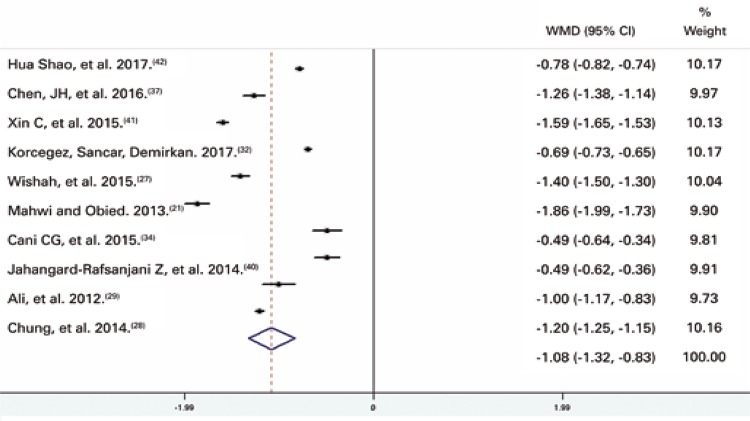
Forest plot

With respect to HbA1c, ten studies^21 , 27 - 29 , 32 , 34 , 36 - 39^ including 715 participants with mean baseline levels of 9.0% were selected. Meta-analysis revealed a mean difference of – 1.07% (95%CI: -1.32; -0.83; p<0.001). The impact of pharmacist interventions on HbA1c reduction in the Control Group is shown in the forest plot ( Figure 3 ).

## Fasting blood glucose levels

Six studies^21 , 27 - 29 , 32 , 39^ involving 457 participants revealed mean fasting blood glucose level reduction of – 29.91mg/dL (95%CI: -43.2; -16.6; p<0.001).

## Triglycerides

Four studies^27 , 29 , 32 , 39^ involving 272 participants were included, with mean TG level reduction of – 19.8mg/dL (95%CI: -36.6; -3.04; p=0.021).

## LDL and HDL

Variations in LDL and HDL levels were non-significant, with mean HDL level increase of 4.43mg/dL (95%CI: 0.16; 8.70; p=0.042), and mean LDL level reduction of – 5.263mg/dL (95%CI: -10.7; 0.18; p=0.058).^27 , 29 , 32 , 39^

## Systolic and diastolic blood pressure

Only SBP differed significantly (mean reduction, - 4.65mmHg; 95%CI: -8.9; -0.4; p=0.032) across all four studies included. Mean reduction in DBP (–1.81mmHg; 95%CI: -3.7; 0.1; p=0.065) was non-significant.^29 , 32 , 37 , 39^

## Frequency of interventions

Six studies involving frequent interventions (monthly or every two months) were included, with mean baseline HbA1c of 8.9%.^21 , 29 , 32 , 36 , 37 , 39^ Mean reduction of – 1.01% (p<0.001) was observed (95%CI: -1.2; -0.7). Mean reduction of – 1.17% (p<0.001; 95%CI: - 1.4; -0.8) was also observed in four studies that failed to report intervals between interventions or reporting intervention interval of three months.^27 , 28 , 34 , 38^ However, mean baseline HbA1c in this group was 9.48%.

## DISCUSSION

According to meta-analysis results, all pharmacist interventions promoted significant reduction of SBP and HbA1c, fasting blood glucose, TG and HDL levels, with no impact on LDL levels or DBP. Santschi et al *.,* ^41^ conducted a meta-analysis of 39 randomized clinical trials with 14,224 participants with high cardiovascular risk (hypertension, dyslipidemia, diabetes, smoking and obesity) and observed SBP (− 7.6mmHg; 95%CI: -9.0; -6.3; I^2^ *=* 67%) and DBP (− 3.9mmHg; 95%CI: -5.1; 2.8; I^2^ *=* 83%) reduction in response to pharmacist interventions, such as patient education, feedback to medical teams, and identification of drug-related problems. Nonetheless, subgroup analysis to investigate differences between diabetic and non-diabetic participants in that study Santschi et al.,^41^ failed to reveal significant differences in SBP (– 6.4mmHg; 95%CI: -7.8; -5.1; p=0.37) and DBP (– 4.5mmHg; 95%CI: -6.3; -2.8; p=0.51). Results of this meta-analysis are in keeping with findings reported by Santschi et al.,^41^ with significant SBP reduction and no impact on DBP.

Deters et al.,^42^ included six studies in a meta-analysis of 640 participants in randomized clinical trials investigating the impact of pharmaceutical care on type 1 and type 2 diabetes. Mean HbA1c difference of – 0.66% (95%CI: - 0.86; -0.45) and non-significant heterogeneity (I^2^ =7.9%; p=0.3659) were reported. Deters et al.,^42^ also considered the meta-analytic effect of pharmacist interventions and evaluated mean HbA1c differences associated with drug-related problems/medication review (-0.79%) and feedback to medical team (-0.81%), and determined the impact of each intervention on the educational component: diabetes-related complications (-0.60%); knowledge about medications (-0.74%); diet, physical exercise and smoking cessation (-0.66%); self-monitoring of blood glucose analysis (-0.74%); definition of individual targets (-0.81%); adherence (-0.60%) and knowledge about diabetes (-0.54%). This study did not include meta-analysis per intervention, since not all clinical trials selected provided detailed description of components, such as number of patients submitted to a given intervention (educational interventions in particular). Only three of the studies included^27 , 32 , 37^ used the Summary of Diabetes Self-Care Activities (SDSCA) to measure the impact of pharmaceutical care in patient self-care components, such as diet, physical exercise practice, self-monitoring of blood glucose, foot care and smoking cessation.

A meta-analysis of 22 randomized clinical trials involving 1,382 participants with T2DM conducted by Aguiar et al *.,* ^23^ revealed a mean HbA1c reduction of – 0.85% (95%CI: -1.06; -0.65; p<0.0001) and significant (p<0.0001) and substantial (I^2^ =67.3%) heterogeneity. Subgroup analyses were conducted to investigate potential causes of heterogeneity, as follows: country where the study was conducted, type of contact with patients, studies using medication review and frequency of interventions, among others. Low (0% to 40%), non-significant (p>0.10) heterogeneity was observed in studies with the following characteristics: clinical trials conducted in the United States; participants with baseline HbA1c levels ≤9%; community pharmacy settings; lack of educational material provision by pharmacists; pharmacist authorized to alter drug prescriptions; more than one intervention per month and proper randomization. Aguiar et al *.,* ^23^ also observed higher mean HbA1c differences in patients with elevated baseline HbA1c levels. Findings of this study revealed greater HbA1c level reduction in trials with longer intervals between pharmacist interventions. However, those studies reported mean baseline HbA1c levels of 9.48%. Therefore, it can be argued that longer intervals between interventions are not associated with increased reduction of HbA1c levels. Likewise Aguiar et al *.,* ^23^ this study showed that patients with elevated baseline HbA1c levels may benefit more from pharmaceutical care, given the mean baseline HbA1c level of participants included in this meta-analysis was 9.0%.

This study reproduced the analysis conducted by Aguiar et al, *.* ^23^ to investigate potential causes of high heterogeneity between studies. Given the small number of studies (less than ten), meta-regression was not performed; rather, subgroup analysis was repeated using the random effect model according to trial characteristics (participants with baseline HbA1c levels <9%;^27 , 32 , 34 , 36 , 37^ interventions such as drug-related problems^27 , 32 , 34 , 37 , 40^ and medication review *;* ^28 , 29^ frequent interventions -monthly or every 2 months;^21 , 26 , 29 , 30 , 32 , 35 , 40 , 42^ outpatient settings^21 , 26 , 27 , 30 - 35^ and community pharmacy;^29 , 37^ ) per sex ( *i.e* ., studies with larger proportion of males^29 , 34 , 41 , 42^ *versus* studies with larger proportion of females).^21 , 27 , 28 , 32 , 37 , 39^ All analyses revealed high and significant heterogeneity (I^2^ >98%; p<0.001). Analysis with study exclusion was also conducted. One trial included only elderly patients (≥65 years).^38^ This was thought to be potentially related to the high, significant heterogeneity observed; hence, sensitivity analysis excluding the aforementioned study was conducted, with similar results: I^2^ *=* 99.2% (p<0.001). Results were also similar following exclusion of studies with short follow-up^21 , 37^ and inadequate randomization.^32^ It was not possible to determine whether lack of specific tools such as Pharmacotherapy WorkUp for drug-related problems might have contributed to heterogeneity, because only two studies reported the use of this tool for that specific purpose.^30 , 35^ Even if all 15 studies selected for systematic review had been included in this meta-analysis, results would still probably be similar. Significant heterogeneity may have reflected differences in inclusion criteria between trials. HbA1c levels for inclusion of studies in this meta-analysis ranged from 6.5% to 9%, with mean variations in baseline HbA1c levels of 9.0% and 8.7%, in the Intervention and Control Groups, respectively. Insulin use^34 , 36 , 38^ is yet another potential cause of heterogeneity, as well as age and sex proportions, short follow-up^21 , 37^ and small number of participants.^29 , 34^

Given the multiculturalism of studies included in this systematic review and meta-analysis, cultural and ethnic differences may also have impacted on clinical outcomes, as may differences between health systems and educational and socioeconomic characteristics of participants. This analysis included studies conducted in different countries and regions around the world (South America, Europe, Eastern Mediterranean, Middle East and Asia), such as those of Chung et al *.,* ^28^ and Siaw et al *.* ,^33^ conducted in Malaysia and Singapore, respectively, enrolling participants of three different ethnic groups (Chinese, Malayans and Indians). Also worthy of notice are potential hurdles regarding intervention acceptance, which may vary in countries where pharmaceutical care is not well accepted. Aguiar et al *.,* ^35^ were the only authors to describe frequency of interventions and acceptance rates (96.9%) by medical teams.

Methodological limitations of studies included in this meta-analysis must also be accounted for. The significance of pharmaceutical care in multiprofessional settings must be emphasized; however, several studies referred in this analysis described Control Group interventions as “standard care”, with no further specifications. The risk of bias in studies selected for this systematic review and meta-analysis was rated “unclear” regarding almost all aspects considered, as few authors reported allocation concealment methods and blinding of outcome raters. Settings where studies were conducted may have impacted results in some cases. Given the small size of the country in which one of the studies was conducted (Turkish Republic of Northern Cyprus), cross contamination between groups may have occurred.^32^ Therefore, the Control Group may have obtained data from the Intervention Group, even in studies conducted in a single outpatient setting (Hawthorne experience). In one of the studies, participants in the Control Group had access to baseline laboratory tests and may have mitigated the effects of pharmacist interventions by seeking greater medical attention.^30^ Also, selected studies did not involve blinding of clinical pharmacists’ activities, with potential increase in care provided by other healthcare professionals in the Control Group. For example, in one trial the clinical pharmacist was allowed to counsel patients in the Control Group, if required.^34^ Clear description of pharmaceutical care provided to participants in the Intervention Group was seldom given and few studies included SDSCA in the educational key component. Hence, statistical analysis per specific key component could not be performed, supporting results reported by Deters et al.,^42^ Overall, studies included in this meta-analysis reported that pharmacists involved in clinical trials were pharmacotherapy specialists and had been trained in diabetes education. However, data regarding training duration (hours) and healthcare professionals in charge were not always provided. Meta-analysis investigating participant adherence could not be conducted due to the wide variability of methods used to measure outcomes and the lack of standardized measures between studies. Standardization of instruments aimed to assess adherence and enable comparisons of findings across studies is therefore required. Frequency of intervention was not always clearly reported in studies in this analysis; conclusions regarding the ideal frequency of intervention could therefore not be drawn. Studies based on interventions performed every three months (or failing to report intervention intervals) reported greater reduction in HbA1c levels. However, mean baseline levels in this group of studies was greater than 9%, in contrast with less than 9% in studies involving monthly interventions. Hence, greater reduction in HbA1c levels may not be directly related to longer interval between interventions. As suggested by Aguiar et al *.* ,^23^ this analysis supports the hypothesis that patients with higher baseline HbA1c levels in may benefit more from pharmaceutical care.

According to the American Diabetes Association (ADA) and *Sociedade Brasileira de Diabetes* (SBD), treatment of *diabetes mellitus* must be determined by an active multiprofessional team, capable of providing continuing education and good quality of care.^1 , 43^ As regards the pharmaceutical profession, SBD refers the Collegiate Resolution (RDC 44/2009) of the National Health Surveillance Agency (ANVISA - *Agência Nacional de Vigilância Sanitária* ) for delivery of pharmaceutical services – including pharmaceutical care and capillary glucose test,^43^ while the ADA guidelines recommend assurance of rational use of insulin, and careful dosing supervision by pharmacists.^1^ Nevertheless, these guidelines fail to describe other components required for multiprofessional patient care.^1 , 44^ Results of this meta-analysis suggest key components such as drug-related problems/medication review (including feedback to medical teams) *,* patient education and follow-up telephone calls are associated with satisfactory clinical outcomes regarding reduction of HbA1c levels. Tools aimed to assess self-care ( *e.g* . SDSCA)^45^ and adherence ( *e.g* . MMAS-8),^46^ or to identify drug-related problems may be useful in patient follow-up.

This study has some limitations. Not all clinical trials selected for systematic review were included in the meta-analysis due to differences in outcome measure reporting (mean, standard deviation, median, IQR and 95%CI). Only studies expressing measures as mean and standard deviation were included. Also, the objective of this study was to identify recent evidence of pharmacist interventions in T2DM and evaluate related impacts. Studies published between 2012 and 2017 were therefore selected. Extension of this time frame would have allowed the inclusion of a greater number of randomized clinical trials. However, studies published before 2012 would likely not reflect current pharmaceutical practices. Data extraction, review and analysis in this study were performed by a single researcher. Still integrity of data presented in this systematic review with meta-analysis can be guaranteed.

## CONCLUSION

This meta-analysis revealed high, significant heterogeneity between studies. Still, the findings suggest pharmaceutical care-related interventions have significant impacts on reduction of systolic blood pressure, glycated hemoglobin, fasting glucose and triglyceride levels, and on increase of HDL levels. The findings also suggest such interventions do not impact on diastolic blood pressure or LDL levels, and that patients with elevated baseline glycated hemoglobin levels may benefit more from pharmaceutical care. However, more randomized clinical trials with better methodological design are warranted for clearer reporting of pharmaceutical intervention outcomes.

## Appendix

**Appendix A:** . Search strategy

PubMed^**®**^
1. *Diabetes mellitus* , type 2 [mh] (107.405)
2. Type 2 diabetes (144.283)
3. T2DM (13.054)
4. Non insulin dependent *diabetes mellitus* (130.159)
5. NIDDM (120.203)
6. 1 OR 2 OR 3 OR 4 OR 5 (156.111)
7. Pharmaceutical services [mh] (60.102)
8. Pharmaceutical care (86.683)
9. Clinical Pharmacy (71859)
10. Community pharmacy (22.968)
11. Pharmacist* (30.355)
12. 7 OR 8 OR 9 OR 10 OR 11 (170.150)
13. Randomized controlled trial [pt] (441.905)
14. Random* AND Control* (797.089)
15. 13 OR 14 (797.180)
16. 6 AND 12 AND 15 (479)
17. Cochrane Central Register of Controlled Trials filter according to date of publication (57)
**Web of Science**
1. ts= Type 2 diabetes (153.853)
2. ts = T2DM (13.045)
3. ts = Non insulin dependent *diabetes mellitus* (10.421)
4. ts = NIDDM (12.313)
5. 1 OR 2 OR 3 OR 4 (168.261)
6. ts = Pharmaceutical services (4.887)
7. ts = Pharmaceutical care (13.954)
8. ts = Clinical pharmacy (8.159)
9. ts = Community pharmacy (6.730)
10.ts = Pharmacist* (26.322)
11.6 OR 7 OR 8 OR 9 OR 10 (47.569)
12.ts = (Random* AND Control*) (586.584)
13. 5 AND 11 AND 12 (211)
14. Filter according to date of publication (59)
**Cochrane Central Register of Controlled Trials**
1. [mh *Diabetes mellitus* , type 2] (11.714)
2. Type 2 Diabetes (28.274)
3. T2DM (2.910)
4. Non insulin dependent *diabetes mellitus* (11.653)
5. NIDDM (1.115)
6. 1 OR 2 OR 3 OR 4 OR 5 (29.876)
7. [mh “Pharmaceutical services”] (1.698)
8. Pharmaceutical care (4.673)
9. Clinical pharmacy (10.716)
10. Community pharmacy (1.874)
11. Pharmacist* (4.188)
12. 7 OR 8 OR 9 OR 10 OR 11 (16.839)
13. 6 AND 12 (1.941)
14. Trials filtered according to date of publication (69)

**Appendix B:** . Risk of bias

	Selection bias		Performance bias	Detection bias	Attrition bias	Reporting bias	Other biases
						
Reference	Random sequence generation	Allocation concealment	Blinding of participants and professionals	Blinding of outcome raters	Incomplete outcomes	Selective outcome reporting	Other sources of bias
Mahwi et al.^(21)^	Unclear risk of bias	Unclear risk of bias	Unclear risk of bias	Unclear risk of bias	Low risk	Low risk	Unclear risk of bias
Jarab et al.^(26)^	Low risk	Unclear risk of bias	Unclear risk of bias	Unclear risk of bias	Low risk	Low risk	Unclear risk of bias
Wishah et al. ^(27)^	Low risk	Unclear risk of bias	Unclear risk of bias	Unclear risk of bias	High risk	High risk	Unclear risk of bias
Chung et al.^(28)^	Unclear risk of bias	Potential risk of bias	Unclear risk of bias	Unclear risk of bias	Low risk	Low risk	Unclear risk of bias
Ali et al.^(29)^	Low risk	Unclear risk of bias	Unclear risk of bias	Unclear risk of bias	Low risk	Low risk	Unclear risk of bias
Mourão et al.^(30)^	Low risk	Unclear risk of bias	Unclear risk of bias	Unclear risk of bias	Low risk	Low risk	Unclear risk of bias
Chan et al.^(31)^	Low risk	Unclear risk of bias	Unclear risk of bias	Unclear risk of bias	Low risk	Low risk	Unclear risk of bias
Korcegez et al.^(32)^	High risk	Unclear risk of bias	Unclear risk of bias	Unclear risk of bias	Low risk	Low risk	Unclear risk of bias
Siaw et al.^(33)^	Low risk	Low risk	Low risk	Unclear risk of bias	Low risk	Low risk	Low risk
Cani et al.^(34)^	Low risk	Unclear risk of bias	High risk	Unclear risk of bias	Low risk	Low risk	Unclear risk of bias
Aguiar et al.^(35)^	Low risk	Unclear risk of bias	Unclear risk of bias	Low risk	Low risk	Low risk	Low risk
Chen et al.^(36)^	Low risk	Unclear risk of bias	Unclear risk of bias	Unclear risk of bias	Low risk	Low risk	Unclear risk of bias
Jahangard-Rafsanjani et al.^(37)^	Low risk	Unclear risk of bias	Unclear risk of bias	Unclear risk of bias	Low risk	Low risk	Unclear risk of bias
Xin et al.^(38)^	Unclear risk of bias	Unclear risk of bias	Unclear risk of bias	Unclear risk of bias	Low risk	Low risk	Risco de viés incerto
Shao et al.^(39)^	Unclear risk of bias	Unclear risk of bias	Unclear risk of bias	Unclear risk of bias	Low risk	Low risk	Unclear risk of bias
