# Supplementary material for: Pharmaceutical care-based interventions in type 2 diabetes mellitus : a systematic review and meta-analysis of randomized clinical trials
Source: Einstein (Sao Paulo). 2020 Jan 27;18:eRW4686. doi: 10.31744/einstein_journal/2020RW4686 (PMC6986882; doi:10.31744/einstein_journal/2020RW4686)
Supplement: Translation

# Translation: Intervenções farmacêuticas no *diabetes mellitus* tipo 2: uma revisão sistemática e metanálise de ensaios clínicos randomizados

**Authors:** Marcel Nogueira, Leonardo Jun Otuyama, Priscilla Alves Rocha, Vanusa Barbosa Pinto

**Affiliations:** 1 Brasil Hospital das Clínicas, Faculdade de Medicina, Universidade de São Paulo, São Paulo, SP, Brasil.

**Keywords:** *Diabetes mellitus* tipo 2, Assistência farmacêutica, Serviço de farmácia hospitalar, Farmacêuticos

## Abstract

**Objetivo:**

Identificar o impacto das intervenções providenciadas pelo cuidado farmacêutico no *diabetes mellitus* tipo 2.

**Métodos:**

Buscas por ensaios clínicos randomizados controlados foram realizadas nas bases PubMed^®^, Cochrane e *Web of Science* . Foram incluídos estudos publicados entre 2012 e 2017, que avaliaram o impacto do cuidado farmacêutico no *diabetes mellitus* tipo 2. A hemoglobina glicada foi o desfecho primário, e os secundários foram pressão arterial, triglicérides e colesterol. O modelo de efeitos aleatórios foi utilizado na metanálise.

**Resultados:**

Foram incluídos 15 estudos envolvendo 2.325 participantes. A metanálise demonstrou heterogeneidade elevada (I^2^>97%; p<0,001), redução nos níveis de hemoglobina glicada (-1,07%; IC95%: -1,32; -0,83; p<0,001), glicose (-29,91mg/dL; IC95%: -43,2; -16,6; p<0,001), triglicérides (19,8mg/dL; IC95%: -36,6; -3,04; p=0,021), pressão arterial sistólica (-4,65mmHg; IC95%: -8,9; -0,4; p=0,032) e aumento do colesterol HDL (4,43mg/dL; IC95%: 0,16; 8,70; p=0,042).

**Conclusão:**

As intervenções clínicas e educacionais providenciadas pelo cuidado farmacêutico têm impacto significativo no *diabetes mellitus* tipo 2. Ferramentas como o *Summary of Diabetes Self-Care Activities* e a *Morisky Medication Adherence Scale* podem ser úteis no acompanhamento dos pacientes.

## INTRODUÇÃO

O *diabetes mellitus* tipo 2 (DMT2) é caracterizado por hiperglicemia pré e pós-prandial, com insuficiência insulínica relativa, tendo origem na secreção inadequada de insulina com sensibilidade reduzida.^1^ É uma doença crônica, que cresce de forma alarmante em diversos países, e deve ser considerado um grave problema de saúde nas próximas décadas.^2 , 3^ O tipo 2 é a forma mais comum do diabetes e tem aumentado em paralelo às mudanças culturais e sociais. Mundialmente estima-se que 415 milhões de pessoas tenham diabetes, com prevalência de 8,3% entre adultos, e estima-se que este número alcance 592 milhões em 2035. No Brasil, existem 14,3 milhões de adultos com diabetes. Além dos gastos do indivíduo com diabetes, devido ao custo da terapia medicamentosa, a doença também gera impacto econômico aos países e a seus respectivos sistemas de saúde, devido às complicações causadas pela doença.^4^

A educação contínua é útil aos pacientes,^1^ já que o manejo do diabetes é complexo e requer esforços que envolvem o monitoramento glicêmico, a adesão ao tratamento, atividade física e mudanças na dieta.^5^ Para avaliar a efetividade do tratamento, a hemoglobina glicada (HbA1c) é um biomarcador utilizado como padrão-ouro para o controle do diabetes, pois reflete os níveis de glicose dos últimos 120 dias, sendo preditor de complicações crônicas severas.^6^ As diretrizes recomendam que os níveis glicêmicos estejam próximos dos níveis normais, para prevenir ou retardar complicações.^1^ Contudo, a não adesão ao tratamento é comum, pode impactar no controle glicêmico e aumentar a mortalidade.^7^ Um controle glicêmico inadequado aumenta o risco de doenças cardiovasculares, neuropatia, retinopatia, nefropatia e hospitalizações.^6 , 8^ A adesão ao tratamento é um passo crucial para o benefício completo do regime terapêutico. Sabe-se que aproximadamente 20% a 50% dos pacientes com doenças crônicas relatam não ter adesão adequada aos medicamentos, o que compromete a efetividade do tratamento.^9 , 10^ Além disso, aqueles que não demonstram adesão apresentam níveis glicêmicos elevados.^11 , 12^

Existem muitos fatores envolvidos na não adesão ao tratamento, incluindo aspectos sociais e econômicos, a complexidade da farmacoterapia e problemas relacionados às crenças do paciente sobre os medicamentos.^13^ Como especialista em farmacoterapia, o farmacêutico clínico contribui para o cuidado ao paciente, auxiliando-o individualmente ou com outros profissionais de saúde, na projeção e no monitoramento de planos terapêuticos, para melhorar o estado patológico, o tratamento e a adesão, por meio de um processo chamado “cuidado farmacêutico”.^14 , 15^ O cuidado farmacêutico pode ser definido como “a provisão responsável da farmacoterapia a fim de alcançar resultados que melhorem a qualidade de vida dos pacientes”.^16^

Também como parte do cuidado farmacêutico, a identificação de problemas relacionados aos medicamentos ( *drug-related problems* ) é essencial para evitar eventos que podem afetar o resultado do tratamento, por exemplo, a efetividade da farmacoterapia, as reações adversas e a polifarmácia, que frequentemente são comuns em pacientes com diabetes.^17 - 21^ O cuidado farmacêutico também providencia informações sobre os medicamentos, fazendo com que o paciente entenda os riscos e os benefícios da farmacoterapia, melhorando a adesão e o desfecho clínico do tratamento.^13^

Entretanto, ainda é necessário compreender as tomadas de decisões no cuidado dos pacientes com diabetes.^22^ Existe a necessidade de identificar quais intervenções farmacêuticas são aplicáveis à prática clínica atualmente. Além disso, há somente uma metanálise sobre o cuidado farmacêutico no DMT2.^23^ A hipótese do nosso estudo é que o cuidado farmacêutico contribui significativamente para o controle do DMT2, reduzindo principalmente a HbA1c dos pacientes.

## OBJETIVO

Avaliar o impacto das intervenções farmacêuticas no controle glicêmico de pacientes com *diabetes mellitus* tipo 2 e outros desfechos, como pressão arterial, triglicérides e colesterol.

## MÉTODOS

### Estratégia de busca

Inicialmente, uma revisão sistemática de ensaios clínicos randomizados controlados sobre o impacto das intervenções farmacêuticas no manejo do DMT2 foi utilizada como referência para inclusão de alguns estudos e, para encontrar novos estudos, também foi adotada a mesma estratégia de busca utilizada pela referida revisão sistemática.^24^ Foram realizadas buscas nas bases de dados PubMed^®^, *Cochrane Central Register of Controlled Trials* e *Web of Science* . A estratégia de busca encontra-se disponível no apêndice A.

### Data de publicação

Foram selecionados estudos publicados entre janeiro de 2012 e dezembro de 2014. Os resultados das novas buscas foram limitados a publicações de janeiro de 2015 a outubro de 2017.

### Seleção dos estudos

A recomendação *Preferred Reporting Items for Systematic Reviews and Meta-Analyses* (PRISMA) foi utilizada para conduzir a revisão sistemática e metanálise.^25^ Foram selecionados estudos com as seguintes características: com população correspondente a participantes com idade maior ou igual a 18 anos, diagnosticados com DMT2; em que o farmacêutico estivesse inserido na equipe de saúde e apto a realizar o cuidado farmacêutico com intervenções clínicas e/ou educacionais no manejo de participantes com DMT2; que comparassem cuidados usuais da equipe médica, da enfermagem e de farmácias comunitárias; cujo desfecho primário tivesse sido a HbA1c e os secundários pressão arterial sistólica (PAS) e diastólica (PAD), triglicérides (TG), colesterol (lipoproteína de baixa – LDL − e alta – HDL − densidade) e adesão ao tratamento; realizados em clínicas ambulatoriais, hospitais e farmácias comunitárias; e cujo desenho fosse o de ensaios clínicos randomizados controlados prospectivos, publicados em inglês.

Foram excluídos estudos que envolveram exclusivamente participantes com *diabetes mellitus* tipo 1, *diabetes insipidus* e diabetes gestacional; tiveram intervenções baseadas apenas em programas educacionais ou no uso de folhetos; cujo desfecho primário foi a glicose (glicemia de jejum); e estudos retrospectivos e ensaios clínicos não randomizados controlados.

### Avaliação do risco de viés

Foi utilizada a ferramenta de colaboração Cochrane, para avaliar o risco de viés de seleção, performance, detecção, atrito, relato e outros vieses. Os estudos selecionados foram classificados como com baixo risco de viés, alto risco de viés e risco de viés incerto.

### Extração dos dados

Os estudos incluídos foram avaliados individualmente por um único autor. Os dados foram extraídos em formato padrão no Microsoft Excel e consistem em características dos estudos, características dos participantes, intervenções farmacêuticas realizadas e as medidas de desfecho.

### Análise estatística

Todas as análises estatísticas foram realizadas usando o software STATA 13 (Statacorp, Texas, EUA). Devido à heterogeneidade dos estudos incluídos na metanálise, o modelo de efeitos aleatórios ( *random-effects model* ) foi selecionado. O I^2^ foi calculado para avaliar a magnitude entre os estudos, sendo que I^2^ >50% indica heterogeneidade substancial e >75%, heterogeneidade considerável. O teste χ^2^ foi utilizado para avaliar a significância da heterogeneidade (p<0,10).

### Medidas de desfecho

Os resultados das medidas de desfecho foram as alterações entre o grupo intervenção e o controle. A diferença média e o intervalo de confiança de 95% (IC95%) foram utilizados para o desfecho primário (HbA1c) e secundários – glicose (glicemia de jejum), PAS, PAD, TG, colesterol (LDL e HDL). Os resultados foram apresentados com as medidas tradicionais: percentagem para HbA1c; mmHg para PAS e PAD; e mg/dL para TG, LDL e HDL. O nível de significância estatística foi considerado como 5%. Resultados extraídos dos estudos selecionados expressos em mmol/L foram convertidos para mg/dL.

## RESULTADOS

### Seleção dos estudos

Foram extraídos da revisão sistemática utilizada como referência para este estudo 36 ensaios clínicos.^24^ A partir dessa revisão sistemática, 25 ensaios foram excluídos, pois estavam fora do período referido de publicação, sendo 11 elegíveis. Dos estudos elegíveis, quatro não apresentaram HbA1c como desfecho primário. Ao final, sete estudos foram incluídos.^21 , 26 - 31^

A estratégia de busca utilizada nas bases de dados resultou em 185 resultados. Na triagem, sete duplicadas foram removidas. Após a análise dos títulos e dos resumos de 161 artigos, 17 foram selecionados. Destes estudos, cinco foram excluídos, pois os artigos não estavam disponíveis na íntegra; dois não apresentaram a HbA1c como desfecho primário; e dois não foram específicos para DMT2. As buscas nas bases de dados resultaram em oito artigos incluídos. No total, 15 artigos foram incluídos na revisão sistemática e 10 na metanálise ( Figura 1 ).

**Figura 1:** Fluxograma de seleção dos estudos HbA1c: hemoglobina glicada.

### Características dos estudos

Os estudos incluídos na revisão sistemática foram conduzidos em clínicas ambulatoriais e farmácias comunitárias de diferentes países, sendo três realizados no Brasil, três na China, um na Malásia, um no Reino Unido, um em Singapura, um em Taiwan, um no Irã, um no Iraque, dois na Jordânia e um na República Turca de Chipre do Norte. O desenho dos estudos incluídos foi randomizado controlado e um multicêntrico. A duração do acompanhamento variou entre 3 a 12 meses, com média de 7,9 meses. Os ensaios clínicos incluíram participantes com HbA1c entre 6,5% a 9%. A descrição detalhada dos estudos incluídos está representada na tabela 1 .

**Tabela 1:** Características dos estudos incluídos

Referência	Cenário	População	Caracterização da amostra	Tempo de acompanhamento	Intervalo de intervenção	Intervenções farmacêuticas	Controle	Medidas de desfecho clínico
Mahwi et al.^(21)^	Centro de diabetes no Iraque	Diabéticos tipo 2, com idade entre 30 e 80 anos	n=65/65* Perdidos no acompanhamento: 3/4* Idade: 52,0±7,86/53,4±10,81*^†^Sexo: 71,0%/ 67,2%* feminino	3 meses	Mensal	Adesão, *drug-related problems*	Cuidados usuais da equipe médica	HbA1c, glicemia de jejum e *drug-related problems,* adesão
Jarab et al.^(26)^	Ambulatório de diabetes de um hospital escola na Jordânia	Diabéticos tipo 2 com idade ≥18 anos, HbA1c ≥7,5%	n=85/86* Perdidos no acompanhamento: 8/7* Idade: 63,4±10,1/65,3±9,2*^†^Sexo: 42,4%/44,2%* feminino	6 meses	8 semanas (acompanhamento telefônico)	Educação do paciente, *drug-related problems* , acompanhamento telefônico, autocuidado	Cuidados usuais da equipe médica/enfermagem	HbA1c, adesão, pressão arterial, CT, HDL, LDL, TG, IMC, autocuidado (SDSCA)
Wishah et al.^(27)^	Ambulatório de diabetes de um hospital escola na Jordânia	Diabéticos tipo 2 com idade ≥18 anos, HbA1c ≥6,5%, em uso de hipoglicemiante oral	n=52/54* Perdidos no acompanhamento: 2/3* Idade: 52,9±9,6/53,2±11,2*^†^Sexo: 61,5%/51,9%* feminino	6 meses	Não informado	Educação do paciente, *drug- related problems* , autocuidado	Cuidados usuais da equipe médica/enfermagem	HbA1c, pressão arterial, CT, HDL, LDL, TG, IMC, glicemia de jejum, autocuidado (SDSCA)
Chung et al.^(28)^	Hospital escola na Malásia	Diabéticos tipo 2 com idade ≥21 anos e <75 anos, HbA1c ≥8%, em uso de hipoglicemiante oral	n=120/121* Perdidos no acompanhamento: NR Idade: 59,7±9,5/58,5±8,3*^†^Sexo: 58,3%/53,7%* feminino	12 meses	3 a 4 meses com acompanhamento telefônico mensal	Revisão da medicação, educação em diabetes	Serviços usuais de farmácias	HbA1c, glicemia de jejum, adesão
Ali et al.^(29)^	Farmácias comunitárias do Reino Unido	Diabéticos tipo 2 com idade ≥18 anos, HbA1c ≥7%, em uso de hipoglicemiante oral	n=25/23* Perdidos no acompanhamento: 2/0* Idade: 66,4±12,7/66,8±10,2*^†^Sexo: 43,5%/56,5%* masculino	12 meses	Mensal pelos 2 primeiros meses e, após, a cada 3 meses	Revisão da medicação, educação do paciente, encaminhamento para outros profissionais de saúde	Cuidados usuais da equipe médica/enfermagem e farmácias comunitárias	HbA1c, glicemia de jejum, pressão arterial, LDL, HDL, TG, IMC, DQoL, HRQoL, adesão, conhecimentos em diabetes (DKT), SIMS
Mourão et al.^(30)^	Unidades Básicas de Saúde no Brasil	Diabéticos tipo 2 com idade ≥18 anos, HbA1c ≥7%, glicemia pós-prandial ≥180mg/dL, em uso de hipoglicemiante oral	n=65/64* Perdidos no acompanhamento: 12/9* Idade: 60,0±10,2/61,3±9,9*^†^Sexo: 68,0%/66,0%* feminino	12 meses	Mensal	*Drug-related problems* , educação em diabetes	Cuidados usuais	HbA1c, pressão arterial, LDL, HDL, TG, IMC, *drug-related problems*
Chan et al.^(31)^	Clínica de diabetes de um hospital público de Hong Kong	Diabéticos tipo 2 com idade ≥18 anos, HbA1c ≥8%, polifármacia, em uso de hipoglicemiante oral	n=51/54* Perdidos no acompanhamento: 0/0* Idade: 63,2±9,5/61,74±11,2*^†^Gênero: 58,8%/51,9%* masculino	9 meses	Não reportado	Educação do paciente, *drug related problems*	Cuidados usuais da equipe médica	HbA1c, pressão arterial, LDL, HDL, TG, IMC, adesão, risco cardiovascular, custo-efetividasde
Korcegez et al.^(32)^	Ambulatório de diabetes de um hospital público da República Turca de Chipre do Norte	Diabéticos tipo 2 com diagnóstico há pelo 6 meses, HbA1c >7% e uso de hipoglicemiante oral	n= 75/77* Perdidos no acompanhamento: 4/3* Idade: 61,80±10,38/62,22±9,54*^†^Sexo: 77,3%/74,0%* feminino	12 meses	2 meses	Educação do paciente, *drug-related problems,* autocuidado	Cuidados usuais da equipe médica/enfermagem	HbA1c, pressão arterial, CT, HDL, LDL, TG, IMC, glicemia de jejum, circunferência abdominal, autocuidado (SDSCA)
Siaw et al.^(33)^	Quatro ambulatórios médicos em Cingapura	Diabéticos tipo 2 com idade ≥21 anos, HbA1c ≥7%, polifarmácia e múltiplas comorbidades	n= 214/197* Perdidos no acompanhamento: 3/1* Idade: 59,2±8,2/60,1±8,1*^†^Sexo: 52,3%/60,9%* masculino	6 meses	4 a 6 semanas	Educação do paciente, *drug-related problems* , ajuste de dose de insulina baseado em sintomas (SIGN *algorithm* ), acompanhamento telefônico	Cuidados usuais da equipe médica	HbA1c, pressão arterial sistólica, LDL, TG, qualidade de vida (PAID), satisfação com o tratamento (DTSQ), uso de serviços de saúde, análise econômica
Cani et al.^(34)^	Ambulatório de diabetes de um hospital-escola em São Paulo	Diabéticos tipo 2 com idade ≥45 anos, HbA1c ≥8,0%, em uso de insulina	n=37/41* Perdidos no acompanhamento: 3/5* Idade: 61,91±9,58/61,58±8,14*^†^Sexo: 34,0%/36%* masculino	6 meses	Não informado	Educação do paciente, *drug-related problems* , técnica de aplicação de insulina	Cuidados usuais	HbA1c, conhecimento em diabetes, conhecimento sobre medicamentos, adesão, técnica de aplicação de insulina, monitoramento glicêmico, qualidade de vida (QoL).
Aguiar et al.^(35)^	Hospital universitário secundário em São Paulo	Diabéticos tipo 2 com diagnóstico há pelo menos 6 meses, HbA1c ≥7%, idade entre 40 e 79 anos, em uso de antidiabético oral	n=36/37* Perdidos no acompanhamento: 0/0* Idade: 61,1,6±7,9/62,4±8,2*^†^Sexo: 69,4%/64,9%* feminino	12 meses	2 a 6 meses (dependendo dos níveis glicêmicos) e acompanhamento telefônico no intervalo das consultas	Educação do paciente, *drug- related problems* , técnica de aplicação de insulina, acompanhamento telefônico	Cuidados usuais da equipe médica/enfermagem	HbA1c, pressão arterial, LDL, adesão
Chen et al.^(36)^	Nantou Hospital, em Taiwan	Diabéticos tipo 2 com idade ≥65 anos, com HbA1c ≥9%	n=50/50* Perdidos no acompanhamento: NR Idade: 72,16±6,6/72,76 ±5,9*^†^Sexo: 50%/50%* masculino e feminino	6 meses	Acompanhamento telefônico mensal	Educação do paciente, *drug-related problems* , técnica de aplicação de insulina, encaminhamento para outros profissionais, acompanhamento telefônico	Cuidados usuais	HbA1c, glicemia de jejum, percentagem de hospitalizações, análise econômica
Jahangard-Rafsanjani et al.^(37)^	Farmácia comunitária no Teerã, Irã	Diabéticos tipo 2 com HbA1c >7% e uso de hipoglicemiante oral	n=51/50* Perdidos no acompanhamento: 6/10* Idade: 57,6±8,3/55,9±8,7*^†^Sexo: 25%/26% feminino	5 meses	Mensal	Educação do paciente, *drug- related problems* , acompanhamento telefônico, autocuidado	Cuidados usuais da equipe médica.	HbA1c, IMC, pressão arterial, adesão, autocuidado (SDSCA)
Xin et al.^(38)^	Tongde Hospital, na província de Hangzhou, China	Diabéticos tipo 2 com idade ≥18 anos, sem uso de insulina nos últimos 18 meses	n=120/120* Perdidos no acompanhamento: 6/7* Idade: 58,8±14,4/59,2±14,2*^†^Sexo: 51,8%/50,4%* masculino	12 meses	Não informado	Educação do paciente, técnica de aplicação de insulina, acompanhamento telefônico	Cuidados usuais da equipe médica	HbA1c, adesão
Shao et al.^(39)^	Hospital universitário na província de Nanjing, China	Diabéticos tipo 2 com idade ≥18 anos, HbA1c ≥7%, diagnóstico de diabetes há pelo menos 3 meses ou mais com uso de hipoglicemiante oral	n=120/120* Perdidos no acompanhamento: 20/21* Idade: 58,86±10,59/59,20±10,34*^†^Sexo: 51%/475%* masculino	6 meses	2 meses (presencial) e acompanhamento telefônico mensal	Educação do paciente, acompanhamento telefônico	Cuidados usuais da equipe médica	HbA1c, adesão, pressão arterial, CT, HDL, LDL, TG, IMC, glicemia de jejum

* relação entre grupo intervenção e grupo controle;^†^idade em anos (média±desvio padrão).

HbA1c: hemoglobina glicada; CT: colesterol total; HDL: lipoproteína de alta densidade; LDL: lipoproteína de baixa densidade; TG: triglicérides; IMC: índice de massa corporal; SDSCA: *Summary of Diabetes Self-Care Activities Questionnaire* ; NR: não reportado; DQoL: *Diabetes Quality of Life Brief Clinical Inventory* ; HRQoL: *Health-Related Quality of Life;* DKT: *Diabetes Knowledge Test* ; SIMS: *Satisfaction with Information Received About Medicines* ; PAID: *Problem Areas in Diabetes Questionnaire* ; DTSQ: *Diabetes Treatment Satisfaction Questionnaire* ; QoL: *Quality of Life* .

### Risco de viés

Sobre o viés de seleção, a geração de sequência aleatória foi considerada adequada na maioria dos estudos (10/15; 66,7%). Quatro (4/15; 26,7%) não reportaram como os participantes foram randomizados. Houve alto risco de viés em um estudo (1/15; 6,7%), no qual os participantes foram randomizados usando o número de registro do prontuário.^32^ O método utilizado para ocultar a sequência aleatória de randomização não foi descrito em 14 estudos (14/15; 93,4%) e apenas um relatou que auditorias foram realizadas para garantir a ocultação de alocação, pois tratava-se de estudo multicêntrico.^33^

Já com relação ao viés de performance, em nenhum estudo houve cegamento das atividades do farmacêutico. Além disso, em 14 estudos, existiu a possibilidade de troca de informações entre os participantes, porque os ensaios foram conduzidos em um único cenário, exceto o estudo multicêntrico de Siaw et al.^33^ Trata-se de um ensaio foi considerado com alto risco de viés de cegamento de participantes e profissionais, pois os participantes do Grupo Controle puderam solicitar informações ao farmacêutico, quando necessário.^34^

Sobre o viés de detecção, apenas um estudo relatou que os avaliadores foram cegados e, dessa forma, não tiveram conhecimento de qual grupo avaliou os desfechos.^35^ Os demais estudos permaneceram com risco de viés incerto, por não descreverem as medidas utilizadas para avaliação dos desfechos clínicos.

Viés de atrito e viés de relato foram encontrados em apenas um estudo, que permaneceu com alto risco de viés, pois um dos desfechos secundários não foi reportado.^27^

Sobre outros vieses, consideramos apenas dois estudos livres de outras fontes de viés (2/15; 13,4%). Treze estudos incluídos nessa revisão sistemática e metanálise (13/15; 86,7%) foram classificados como risco de viés incerto, pois as limitações apresentadas pelos autores foram insuficientes para julgar se um importante risco de viés poderia impactar nos desfechos dos participantes ( Figura 2 e Apêndice B).

**Figura 2:** Risco de viés

### Características da população

Foram envolvidos nos estudos incluídos nessa revisão sistemática 2.325 participantes. A amostra variou de 36 a 214 participantes. A proporção de participantes do sexo masculino variou de 22% a 75%. A média da idade variou de 52 a 72 anos. A média basal de HbA1c foi de 9,06% no Grupo Intervenção e 8,79% no Grupo Controle. Além do DMT2, a maior parte dos estudos incluídos enfatizou múltiplas comorbidades no desfecho clínico secundário, como dislipidemia, hipertensão e obesidade ( Tabela 1 ).

### Intervenções farmacêuticas

As intervenções foram conduzidas em consultas farmacêuticas presenciais e em alguns estudos, também por acompanhamento telefônico (7/15; 46,7%). Entretanto, nem todos os estudos esclareceram as intervenções realizadas no acompanhamento telefônico. Todos os ensaios incluídos tiveram foco em ações educacionais com o paciente (15/15; 100%), além de ações colaborativas com a equipe médica (14/15; 93,4%). Porém, quando os termos “ *drug-related problems* ” (12/15; 80,0%) ou “ *medication review* ” (2/15; 26,7%) foram utilizados, não houve explicações claras de quais instrumentos foram aplicados. Dois estudos^21 , 31^ consideraram a frequência de *drug-related problems* como desfecho clínico dos participantes, e apenas dois^30 , 35^ descreveram que o *Pharmacotherapy WorkUp* ^40^ foi utilizado como ferramenta para tal finalidade.

No acompanhamento dos participantes, as ferramentas “ *drug related problems* ” e *“medication review”* foram utilizadas para identificar e solucionar problemas relacionados aos medicamentos. Tais ferramentas incluem componentes-chave de intervenções farmacêuticas como: *Feedback* – recomendações sobre o tratamento farmacológico foram direcionadas à equipe médica com o objetivo de solucionar os problemas identificados e otimizar a farmacoterapia (adicionar, substituir ou suspender medicamentos, além de ajustar doses). Acompanhamento telefônico – orientação farmacêutica por telefone e avaliação de eventos adversos. Educação do paciente – intervenções educacionais sobre o diabetes e o tratamento também foram realizadas para melhorar a adesão dos participantes, como o fornecimento de informações sobre os medicamentos, reações adversas, forma de administração e armazenamento (especialmente de insulina), treinamento no reconhecimento e na correção de hipoglicemia, alterações no estilo de vida (cessação do tabagismo, etilismo, dieta adequada e inspeção dos pés), promoção ao autocuidado (monitoramento glicêmico). Todos os pacientes receberam instruções verbais e, em alguns casos, também foram utilizados materiais educativos, como folhetos.

Em um estudo, o farmacêutico clínico teve autoridade para realizar ajustes nas doses de insulina em pacientes virgens de tratamento, baseando-se em sinais/sintomas de hipoglicemia. Para isso, um algoritmo foi validado.^33^

Em dois estudos^29 , 36^ (2/15, 26,7%), os participantes foram encaminhados a outros profissionais de saúde como nutricionistas e enfermeiros.

O Grupo Controle consistiu nos cuidados usuais realizados apenas pelas unidades ambulatoriais e farmácias comunitárias, sem as intervenções do farmacêutico clínico ou educação em diabetes providenciada por outros profissionais de saúde, como médicos e enfermeiros ( Tabela 1 ).

### Adesão

A adesão ao tratamento foi considerada um desfecho clínico em dez estudos. Para avaliar a adesão dos participantes, foi utilizada a *Morisky Medication Adherence Scale* (MMAS-8 e MMAS-4) na linha de base do início do estudo e no final do acompanhamento. Em todos os estudos, foram reportadas melhoras significativas da adesão no Grupo Intervenção *versus* Grupo Controle (p<0,05).

### Metanálise

#### Análise da heterogeneidade

Dez estudos foram incluídos na metanálise.^21 , 27 - 29 , 32 , 34 , 36 - 39^ Todos apresentaram heterogeneidade elevada, com o I^2^ entre 97% e 99% (p<0,001) para todos os desfechos. Mesmo com a considerável heterogeneidade, os resultados demonstraram que o cuidado farmacêutico é eficaz em reduzir os níveis de HbA1c, glicose (glicemia de jejum), TG e PAS, e, também, de elevar o HDL. Entretanto, não houve diferenças estatisticamente significativas nos desfechos de LDL e PAD.

## Níveis de hemoglobina glicada

Para o desfecho de HbA1c, foram selecionados dez estudos^21 , 27 - 29 , 32 , 34 , 36 - 39^ que incluíram 715 participantes com média basal de 9,0%, e a metanálise resultou em diferença média de -1,07% (IC95%: -1,32; -0,83; p<0,001). O impacto das intervenções farmacêuticas comparado ao Grupo Controle na redução de HbA1c é demonstrado no gráfico de floresta da figura 3 .

**Figura 3:** Forest plot

## Glicose (glicemia de jejum)

Seis estudos^21 , 27 - 29 , 32 , 39^ realizados em um total de 457 participantes demonstraram redução média de -29,91mg/dL (IC95%: -43,2; -16,6; p<0,001).

## Triglicérides

Foram incluídos quatro estudos^27 , 29 , 32 , 39^ conduzidos em 272 participantes, e houve redução média de -19,8mg/dL (IC95%: -36,6; -3,04; p=0,021).

## LDL e HDL

Houve aumento médio de 4,43mg/dL (IC95%: 0,16; 8,70; p=0,042) para o HDL, e, para o desfecho de LDL, observou-se redução média de -5,263mg/dL (IC95%: -10,7; 0,18; p=0,058), não sendo estatisticamente significativo.^27 , 29 , 32 , 39^

## Pressão arterial sistólica e pressão arterial diastólica

Em quatro estudos incluídos, houve diferença estatisticamente significativa apenas para o desfecho de PAS, com redução média de -4,65mmHg (IC95%: -8,9; -0,4; p =0,032). Para o desfecho de PAD, foi observada redução de -1,81mmHg (IC95%: -3,7; 0,1; p=0,065), não sendo estatisticamente significativa.^29 , 32 , 37 , 39^

## Frequência de intervenções

Foram analisados seis estudos que fizeram intervenções frequentes (mensais ou a cada dois meses), com média de HbA1c basal de 8,9%.^21 , 29 , 32 , 36 , 37 , 39^ Houve uma redução média de -1,01% (p<0,001) IC95% (-1.2; -0,7). Também observamos uma redução média de -1,17% (p<0,001) IC95% (-1,4; -0,8) para quatro estudos que não especificaram o intervalo de intervenções ou que foram realizadas a cada três meses.^27 , 28 , 34 , 38^ Porém, a média de HbA1c basal neste grupo foi de 9,48%.

## DISCUSSÃO

Os resultados da metanálise demonstraram que todas as intervenções farmacêuticas reduziram de forma significativa os níveis de HbA1c, glicemia de jejum, TG, PAS e HDL, não tendo impacto na LDL e nem na PAD. Santschi et al.,^41^ realizaram metanálise de 39 ensaios clínicos randomizados com 14.224 participantes com risco cardiovascular elevado (hipertensão, dislipidemia, diabetes, tabagismo e obesidade) e observou-se que as intervenções farmacêuticas, como educação do paciente, *feedback* à equipe médica e identificação de problemas relacionados aos medicamentos reduziram a PAS (−7,6mmHg; IC95%: -9,0; -6,3; I^2^ *=* 67%) e PAD (−3,9mmHg; IC95%: -5,1; -2,8; I^2^ *=* 83%). Porém Santschi et al.,^41^ também realizaram análise de subgrupo que verificou se houve diferença estatisticamente significativa entre grupos de estudos de participantes com diabetes *versus* estudos com participantes sem diabetes. O resultado não revelou diferença estatisticamente significativa entre os dois grupos de estudos: PAS -6,4mmHg (IC95%: -7,8; -5,1; p=0,37) e PAD -4,5mmHg (IC95%: -6,3; -2,8; p=0,51). Corroboramos o que foi apresentado por Santschi et al.,^41^ pois também observamos redução estatisticamente significativa na PAS, mesmo sem impacto na redução da PAD em nossa metanálise.

Deters et al.,^42^ incluíram seis estudos em metanálise de 640 participantes de ensaios clínicos randomizados em diabetes tipos 1 e 2, que avaliou o impacto do cuidado farmacêutico. Houve diferença média de HbA1c de - 0,66% (IC95%: -0,86; -0,45) com heterogeneidade insignificante (I^2^ =7,9%; p=0,3659). Uma análise do efeito metanalítico das intervenções farmacêuticas também foi executada por Deters et al.,^42^ que, além de avaliarem a diferença média de HbA1c relacionada ao *drug-related problems/medication review* (-0,79%) e ao *feedback* à equipe médica (-0,81%), especificaram o impacto de cada intervenção realizada no componente educacional: complicações relacionadas ao diabetes (-0,60%); conhecimentos sobre os medicamentos (-0,74%); dieta, exercícios físicos e cessação do tabagismo (-0,66%); análise do automonitoramento glicêmico (-0,74%); definição de metas individuais (-0,81%); adesão (-0,60%); e conhecimentos em diabetes (-0,54%). Não realizamos metanálise por intervenções farmacêuticas, pois nem todos os ensaios clínicos incluídos em nosso estudo especificaram os componentes detalhadamente, como o número de pacientes submetidos a tipos de intervenções, especialmente as educacionais. Apenas três estudos incluídos^27 , 32 , 37^ utilizaram o *Summary of Diabetes Self-Care Activities* (SDSCA) para mensurar o impacto do cuidado farmacêutico no autocuidado dos participantes em componentes como dieta, prática de exercícios físicos, automonitoramento glicêmico, cuidados com os pés e cessação do tabagismo.

Aguiar et al.,^23^ conduziram metanálise de 22 ensaios clínicos randomizados em 1.382 participantes com DMT2, que revelou redução média para HbA1c de -0,85% (IC95%: -1,06; -0,65; p<0,0001) e heterogeneidade significativa (p<0,0001) e substancial (I^2^ =67,3%). Para explorar as causas de heterogeneidade, análises de subgrupos foram executadas da seguinte forma: país em que o estudo foi conduzido; forma de contato com o paciente; estudos que utilizaram o *medication review;* frequência de intervenções, entre outras. Foi observado que não houve heterogeneidade elevada (0% a 40%) e não significativa (p>0,10) nos estudos com as seguintes características: ensaios clínicos conduzidos nos Estados Unidos; participantes com HbA1c basal ≤9%; farmácias comunitárias como cenário ; sem uso de materiais de suporte educacional fornecidos pelo farmacêutico; farmacêutico com autonomia para alterar a prescrição de medicamentos; frequência de intervenções mais de uma vez ao mês e processo de randomização adequado. Aguiar et al.,^23^ também observaram que as diferenças médias nos níveis de HbA1c aumentam com HbA1c basal elevada. Nossos resultados demonstraram também maior redução de HbA1c em estudos que tiveram intervalo de tempo mais longo nas intervenções farmacêuticas. Entretanto, esses estudos apresentaram média basal de HbA1c de 9,48%, e, por esse motivo, podemos supor que um maior intervalo de intervenções não está associado com maior redução de HbA1c. Com isso, assim como Aguiar et al.,^23^ afirmamos que pacientes com níveis basais elevados de HbA1c podem ter melhores benefícios do cuidado farmacêutico, já que a média basal de HbA1c dos participantes incluídos em nossa metanálise foi de 9,0%.

Para explorar as possíveis causas da heterogeneidade elevada entre os estudos, reproduzimos análise semelhante à realizada por Aguiar et al.^23^ Não realizamos metarregressão por conta do número pequeno de estudos (menos que dez) e, dessa forma, conduzimos novamente a análise de subgrupos usando o modelo de efeitos aleatórios, de acordo com as características de cada ensaio: participantes com valores basais de HbA1c <9%;^27 , 32 , 34 , 36 , 37^ intervenções como *drug-related problems* ^27 , 32 , 34 , 37 , 40^ e *medication review;* ^28 , 29^ intervenções frequentes (mensais ou a cada 2 meses);^21 , 26 , 29 , 30 , 32 , 35 , 40 , 42^ por cenários ambulatorial^21 , 26 , 27 , 30 - 35^ e da farmácia comunitária;^29 , 37^ por sexo, ou seja, estudos com maior proporção de homens^29 , 34 , 41 , 42^ contra estudos com maior proporção de mulheres.^21 , 27 , 28 , 32 , 37 , 39^ Todas as análises demonstraram heterogeneidade elevada e significativa (I^2^ >98%; p<0,001). Realizamos também análises por exclusão de estudos. Um dos ensaios incluiu apenas idosos (≥65 anos)^38^ e levantando a hipótese de que isso poderia ter relação com a heterogeneidade elevada e significativa, a análise de sensibilidade também foi executada sem a inclusão desse estudo e não mostrou resultado diferente: I^2^ *=* 99,2% (p<0,001). Também não encontramos resultados diferentes ao excluir estudos que tiveram curto período de acompanhamento^21 , 37^ e processo de randomização inadequado.^32^ Não foi possível identificar se a ausência de uma ferramenta específica, como o *Pharmacotherapy WorkUp* , utilizada para *drug-related* problems, poderia contribuir para a heterogeneidade, pois somente dois ensaios clínicos declararam utilizá-la para essa finalidade.^30 , 35^ Mesmo se os 15 estudos selecionados para revisão sistemática fossem incluídos em nossa metanálise, provavelmente, não encontraríamos resultado diferente. Entre as prováveis causas da heterogeneidade significativa, estão os diferentes critérios de inclusão dos ensaios conduzidos. Os níveis de HbA1c exigidos para inclusão dos participantes nos estudos incluídos foi de 6,5% a 9%. Isso resultou em variação média de HbA1c basal de 9,0% no Grupo Intervenção e 8,7% no Grupo Controle. O uso de insulina^34 , 36 , 38^ também pode ser considerado provável causa, assim como a proporção de idade e sexo, o período curto de acompanhamento^21 , 37^ e o número pequeno de participantes.^29 , 34^

Devido ao multiculturalismo dos estudos incluídos em nossa revisão sistemática com metanálise, é possível que as diferenças culturais e étnicas também tenham influenciado no desfecho clínico, além das diferenças entre os sistemas de saúde, nível educacional e socioeconômico dos participantes. Incluímos estudos de diferentes países e regiões do mundo (América do Sul, Europa, Mediterrâneo Oriental, Oriente Médio e Ásia), como, por exemplo, os estudos de Chung et al.,^28^ e Siaw et al.,^33^ realizados na Malásia e Cingapura, respectivamente. Esses ensaios envolveram participantes de três grupos étnicos diferentes (chineses, malaios e indianos). Também é necessário considerar possíveis obstáculos sob a aceitação das intervenções, que podem variar em países onde o cuidado farmacêutico não é bem aceito. Somente Aguiar et al.,^35^ descreveram a frequência das intervenções realizadas e a taxa de aceitação (96,9%) pela equipe médica.

Além disso, temos que considerar todas as limitações metodológicas dos estudos incluídos em nossa metanálise. Ressaltamos que o cuidado farmacêutico é importante dentro de um contexto multiprofissional, embora diversos estudos aqui citados relataram as intervenções do Grupo Controle como “cuidados usuais”, sem maiores especificações. O risco de viés dos estudos selecionados para essa revisão sistemática com metanálise prevaleceu como incerto em quase todos os aspectos avaliados, já que poucos autores reportaram os métodos de ocultação de alocação e de cegamento dos avaliadores de desfecho. Alguns cenários utilizados para a condução dos estudos podem ter influenciado nos resultados. Devido ao pequeno país em que um dos estudos foi realizado (República Turca de Chipre do Norte), houve o risco de contaminação cruzada entre os grupos.^32^ Dessa forma, é possível que o Grupo Controle tenha obtido informações com o Grupo Intervenção, até mesmo em estudos conduzidos em um único cenário ambulatorial (experiência de *Hawthorne* ). Em um dos estudos, os participantes do Grupo Controle tiveram acesso aos exames laboratoriais na linha de base e, dessa forma, ao solicitar maior atenção médica, poderiam minimizar os efeitos da intervenção farmacêutica.^30^ Também não houve cegamento das atividades do farmacêutico clínico nos estudos selecionados e, com isso, possivelmente houve aumento no cuidado de outros profissionais de saúde no Grupo Controle. Por exemplo, em um ensaio, o farmacêutico clínico estava disponível para orientar pacientes do grupo controle, se necessário.^34^ Poucos autores descreveram claramente o cuidado farmacêutico recebido pelos participantes do Grupo Intervenção. Também, poucos ensaios utilizaram o SDSCA no componente-chave educacional. Por isso, não foi possível executar análise estatística específica por componente-chave, contribuindo com os resultados apresentados por Deters et al.^42^ No geral, todos os estudos incluídos na metanálise declararam que os farmacêuticos envolvidos nos ensaios clínicos eram especialistas em farmacoterapia e receberam treinamento para serem educadores em diabetes. Porém, nem todos especificaram a duração do treinamento (em horas) e qual profissional de saúde foi providenciado para treinar os farmacêuticos. Também não realizamos metanálise de adesão dos participantes, pois a ampla variabilidade de métodos utilizados para mensurar os resultados e a falta de medidas padronizadas entre os estudos não permitiram avaliação. Isso demonstra que é necessário padronizar os instrumentos utilizados para avaliar a adesão e, dessa forma, possibilitar a comparação dos resultados de diferentes estudos. Nosso resultado também não permitiu concluir com qual frequência as intervenções farmacêuticas devem ser realizadas, pois nem todos os autores reportaram com clareza essas informações. Foi observado que o grupo de estudos que realizou intervenções a cada 3 meses (ou que não relatou o intervalo de intervenções) apresentou maior redução nos níveis de HbA1c. Porém, a média basal nesse grupo foi maior que 9% e a do grupo de estudos que realizou intervenções mensais, menor que 9%. Portanto, é provável que um maior efeito na redução de HbA1c não tenha relação direta com intervalo maior de intervenções, pois, assim como Aguiar et al.,^23^ corroboramos que os pacientes com maiores níveis basais de HbA1c terão melhor benefício do cuidado farmacêutico.

As diretrizes da *American Diabetes Association* (ADA) e da Sociedade Brasileira de Diabetes (SBD) afirmam que o processo terapêutico no *diabetes mellitus* deve ser estabelecido por equipe multiprofissional ativa, capacitada para providenciar educação contínua, além de assistência de qualidade.^1 , 43^ No âmbito farmacêutico, a SBD cita a Resolução da Diretoria Colegiada (RDC) 44/2009 da Agência Nacional de Vigilância Sanitária (Anvisa), que normatizou a prestação de serviços farmacêuticos – entre eles, o cuidado farmacêutico e a glicemia capilar.^43^ Por outro lado, as diretrizes da ADA recomendam a garantia do uso racional de insulina e da supervisão meticulosa do farmacêutico sobre a dose administrada.^1^ Entretanto, essas diretrizes não especificam outros componentes necessários para prestar o cuidado ao paciente no âmbito multiprofissional.^1 , 44^ Os resultados da nossa metanálise sugerem que componentes-chave, como *drug-related problems/medication review* (incluindo o *feedback* à equipe médica) *,* educação do paciente e acompanhamento telefônico são intervenções que demonstraram desfecho clínico satisfatório na redução de HbA1c dos participantes. O uso de ferramentas que avaliam o autocuidado, como o SDSCA,^45^ a adesão, como o MMAS-8,^46^ e que ajudam a identificar problemas relacionados aos medicamentos ( *drug-related problems* ) podem ser úteis no acompanhamento dos pacientes.

Nosso estudo também apresenta algumas limitações. Nem todos os ensaios clínicos selecionados para a revisão sistemática foram incluídos na metanálise, por apresentarem diferentes medidas nos relatos de desfecho clínico (média, desvio padrão, mediana, IQR e IC95%). Por esse motivo, incluímos apenas estudos cujas medidas foram descritas como média e desvio padrão. Além disso, nosso objetivo foi identificar evidências mais recentes das intervenções farmacêuticas no DMT2 e avaliar seu impacto. Por isso, selecionamos estudos publicados nos entre 2012 e 2017. Um período de referência maior permitiria incluir mais ensaios clínicos randomizados. Porém, provavelmente, a inclusão de estudos anteriores ao ano de 2012 não refletiria a prática farmacêutica atual. Ainda que apenas um único autor tenha extraído, revisado e analisado todos os dados, garantimos a integridade dos dados apresentados nessa revisão sistemática com metanálise.

## CONCLUSÃO

Embora nossa metanálise tenha demonstrado heterogeneidade elevada e significativa, nossos resultados sugerem que as intervenções providenciadas pelo cuidado farmacêutico têm impacto significativo na redução de hemoglobina glicada, glicemia de jejum, triglicérides, pressão arterial sistólica e no aumento dos níveis do HDL. Nossos resultados também sugerem que essas intervenções não geram impacto na pressão arterial diastólica e na LDL. Além disso, pacientes com níveis basais elevados de hemoglobina glicada devem ter maiores benefícios do cuidado farmacêutico. No entanto, mais ensaios clínicos randomizados devem ser realizados, com melhor delineamento metodológico, para que seja possível reportar com maior clareza as intervenções farmacêuticas.

## Apêndice

**Apêndice A:** . Estratégia de busca

PubMed®
1. *Diabetes mellitus* , type 2 [mh] (107.405)
2. Type 2 diabetes (144.283)
3. T2DM (13.054)
4. Non insulin dependent *diabetes mellitus* (130.159)
5. NIDDM (120.203)
6. 1 OR 2 OR 3 OR 4 OR 5 (156.111)
7. Pharmaceutical services [mh] (60.102)
8. Pharmaceutical care (86.683)
9. Clinical Pharmacy (71859)
10. Community pharmacy (22.968)
11. Pharmacist* (30.355)
12. 7 OR 8 OR 9 OR 10 OR 11 (170.150)
13. Randomized controlled trial [pt] (441.905)
14. Random* AND Control* (797.089)
15. 13 OR 14 (797.180)
16. 6 AND 12 AND 15 (479)
17. Filtro da *Cochrane Central Register of Controlled Trials* por data de publicação (57)
***Web of Science***
1. ts= Type 2 diabetes (153.853)
2. ts = T2DM (13.045)
3. ts = Non insulin dependent *diabetes mellitus* (10.421)
4. ts = NIDDM (12.313)
5. 1 OR 2 OR 3 OR 4 (168.261)
6. ts = Pharmaceutical services (4.887)
7. ts = Pharmaceutical care (13.954)
8. ts = Clinical pharmacy (8.159)
9. ts = Community pharmacy (6.730)
10.ts = Pharmacist* (26.322)
11.6 OR 7 OR 8 OR 9 OR 10 (47.569)
12.ts = (Random* AND Control*) (586.584)
13. 5 AND 11 AND 12 (211)
14. Com filtro por data de publicação (59)
***Cochrane Central Register of Controlled Trials***
1. [mh *Diabetes mellitus* , type 2] (11.714)
2. Type 2 Diabetes (28.274)
3. T2DM (2.910)
4. Non insulin dependent *diabetes mellitus* (11.653)
5. NIDDM (1.115)
6. 1 OR 2 OR 3 OR 4 OR 5 (29.876)
7. [mh “Pharmaceutical services”] (1.698)
8. Pharmaceutical care (4.673)
9. Clinical pharmacy (10.716)
10. Community pharmacy (1.874)
11. Pharmacist* (4.188)
12. 7 OR 8 OR 9 OR 10 OR 11 (16.839)
13. 6 AND 12 (1.941)
14. Trials com filtro por data de publicação (69)

**Apêndice B:** . Risco de viés

	Viés de seleção		Viés de performance	Viés de detecção	Viés de atrito	Viés de relato	Outros vieses
						
Referência	Geração de sequência aleatória	Ocultação de alocação	Cegamento de participantes e profissionais	Cegamento de avaliadores de desfecho	Desfechos incompletos	Relato de desfecho seletivo	Outras fontes de viés
Mahwi et al.^(21)^	Risco de viés incerto	Risco de viés incerto	Risco de viés incerto	Risco de viés incerto	Baixo risco	Baixo risco	Risco de viés incerto
Jarab et al.^(26)^	Baixo risco	Risco de viés incerto	Risco de viés incerto	Risco de viés incerto	Baixo risco	Baixo risco	Risco de viés incerto
Wishah et al.^(27)^	Baixo risco	Risco de viés incerto	Risco de viés incerto	Risco de viés incerto	Alto risco	Alto risco	Risco de viés incerto
Chung et al.^(28)^	Risco de viés incerto	Risco de viés incerto	Risco de viés incerto	Risco de viés incerto	Baixo risco	Baixo risco	Risco de viés incerto
Ali et al.^(29)^	Baixo risco	Risco de viés incerto	Risco de viés incerto	Risco de viés incerto	Baixo risco	Baixo risco	Risco de viés incerto
Mourão et al.^(30)^	Baixo risco	Risco de viés incerto	Risco de viés incerto	Risco de viés incerto	Baixo risco	Baixo risco	Risco de viés incerto
Chan et al.^(31)^	Baixo risco	Risco de viés incerto	Risco de viés incerto	Risco de viés incerto	Baixo risco	Baixo risco	Risco de viés incerto
Korcegez et al.^(32)^	Alto risco	Risco de viés incerto	Risco de viés incerto	Risco de viés incerto	Baixo risco	Baixo risco	Risco de viés incerto
Siaw et al.^(33)^	Baixo risco	Baixo risco	Baixo risco	Risco de viés incerto	Baixo risco	Baixo risco	Baixo risco
Cani et al.^(34)^	Baixo risco	Risco de viés incerto	Alto risco	Risco de viés incerto	Baixo risco	Baixo risco	Risco de viés incerto
Aguiar et al.^(35)^	Baixo risco	Risco de viés incerto	Risco de viés incerto	Baixo risco	Baixo risco	Baixo risco	Baixo risco
Chen et al.^(36)^	Baixo risco	Risco de viés incerto	Risco de viés incerto	Risco de viés incerto	Baixo risco	Baixo risco	Risco de viés incerto
Jahangard-Rafsanjani et al.^(37)^	Baixo risco	Risco de viés incerto	Risco de viés incerto	Risco de viés incerto	Baixo risco	Baixo risco	Risco de viés incerto
Xin et al.^(38)^	Risco de viés incerto	Risco de viés incerto	Risco de viés incerto	Risco de viés incerto	Baixo risco	Baixo risco	Risco de viés incerto
Shao et al.^(39)^	Risco de viés incerto	Risco de viés incerto	Risco de viés incerto	Risco de viés incerto	Baixo risco	Baixo risco	Risco de viés incerto
